# Peptide-Recombinant VP6 Protein Based Enzyme Immunoassay for the Detection of Group A Rotaviruses in Multiple Host Species

**DOI:** 10.1371/journal.pone.0159027

**Published:** 2016-07-08

**Authors:** Naveen Kumar, Yashpal Singh Malik, Satish Kumar, Kuldeep Sharma, Subhankar Sircar, Sharad Saurabh, Baldev R. Gulati, Neeraj Singh, Arvind Kumar Singh, Vinay G. Joshi, Krisztian Banyai, Kuldeep Dhama

**Affiliations:** 1 Biological Standardization Division, Indian Veterinary Research Institute (IVRI), Izatnagar, Uttar Pradesh, India; 2 Division of Animal Biotechnology, Indian Veterinary Research Institute (IVRI), Izatnagar, Uttar Pradesh, India; 3 National Institute of Research in Tribal Health, Jabalpur, Madhya Pradesh, India; 4 National Research Center on Equines (NRCE), Hisar, Haryana, India; 5 Institute for Veterinary Medical Research, Centre for Agricultural Research, Hungarian Academy of Sciences, Hungáriakrt. 21, Budapest, Hungary; 6 Division of Veterinary Immunology, Indian Veterinary Research Institute (IVRI), Izatnagar, Uttar Pradesh, India; CNR, ITALY

## Abstract

We developed a novel enzyme immunoassay for the detection of group A rotavirus (RVA) antigen in fecal samples of multiple host species. The assay is based on the detection of conserved VP6 protein using anti-recombinant VP6 antibodies as capture antibodies and anti-multiple antigenic peptide (identified and constructed from highly immunodominant epitopes within VP6 protein) antibodies as detector antibodies. The clinical utility of the assay was evaluated using a panel of 914 diarrhoeic fecal samples from four different host species (bovine, porcine, poultry and human) collected from diverse geographical locations in India. Using VP6- based reverse transcription-polymerase chain reaction (RT-PCR) as the gold standard, we found that the diagnostic sensitivity (DSn) and specificity (DSp) of the new assay was high [bovine (DSn = 94.2% & DSp = 100%); porcine (DSn = 94.6% & DSp = 93.3%); poultry (DSn = 74.2% & DSp = 97.7%) and human (DSn = 82.1% & DSp = 98.7%)]. The concordance with RT-PCR was also high [weighted kappa (k) = 0.831–0.956 at 95% CI = 0.711–1.0] as compared to RNA-polyacrylamide gel electrophoresis (RNA-PAGE). The performance characteristics of the new immunoassay were comparable to those of the two commercially available ELISA kits. Our results suggest that this peptide-recombinant protein based assay may serve as a preliminary assay for epidemiological surveillance of RVA antigen and for evaluation of vaccine effectiveness especially in low and middle income settings.

## Introduction

Rotaviruses (RVs) belong to the family *Reoviridae* and are a leading cause of diarrhea in humans and animals worldwide. For example, RVs account for approximately one fourth of global mortality in Indian children annually [1–3]. The genome consists of 11 dsRNA segments, which encode six structural proteins (VP1–4, VP6 and VP7) and five/six non-structural proteins (NSP1–NSP5/6) [4]. RVs are classified into at least nine distinct serological species or groups (A to I), of which group A rotaviruses (RVAs) are frequently associated with acute diarrhea [1,2,5]. Commonly used dual classification system for RVAs designate P- and G-genotypes to the genes coding for outer most proteins VP4 and VP7, respectively. At least 27 G and 37 P genotypes of RVAs have been identified in humans and animals, reflecting huge genetic diversity among RVs [2,6].

Rapid and accurate diagnosis of RVAs remains a major hurdle, especially in the low and middle income countries. Currently, the diagnosis of RVAs relies on the detection of either the virus antigen or its genome. Although highly sensitive, the reverse transcription- polymerase chain reaction (RT-PCR) is costly and is not affordable for conducting surveillance studies, especially in poor countries [7–8]. On the other hand, electropherotyping and latex agglutination tests are cost effective, but their low sensitivity and specificity limits their use on a large scale [9–10]. In this context, enzyme-linked immunosorbent assay (ELISA) is considered an ideal diagnostic tool because it is known to be effective and economical for RVA surveillance studies. Unfortunately, the use of polyclonal serum against whole virus antigen makes this test prone to false reactions. Synthetic peptides prepared from antigenic regions of the virus may provide an alternative to the whole virus and has several other advantages, e.g., it is stable at pH 2–9, avoids risk of infection, instant manufacturing on a large scale, and durable storage [11–12].

Despite the huge genetic diversity of RVAs, the sole structural protein of the middle of three capsid layers (VP6) is highly immunogenic and conserved among RVAs [13], which makes it preferable to be used as a universal diagnostic reagent. However, tracing the regions within the VP6 protein that are highly reactive with anti-RVA antibodies is imperative for effective use of peptides in a diagnostic assay. The anti-peptide antibodies (usually targeted against one or two epitopes) could serve as homogenous antibodies comparable to monoclonal antibodies, thereby increasing the specificity as well as reducing the cost and clumsy procedure employed in monoclonal antibody production [14]. Further, engaging multiple antigenic peptides (MAPs) may overcome the problem of low titer anti-peptide serum [15–16]. MAPs have essentially three parts viz. an amino acid (alanine/cysteine) linked to a solid support or resin, an inner lysine core matrix, and a surface layer of four or eight peptides of the same/different sequences attached to the core matrix. Attaching the four or eight peptides over the lysine core matrix increases total molecular weight and mimics closely the native antigenic sites present on the virus surface. Owing to these advantages, anti-peptide antibodies have been used for serodiagnosis of hepatitis C virus [17], infectious bronchitis virus [18], infectious bursal disease virus [19–20] and peste-des-petits ruminants virus [21].

**Fig 1:**
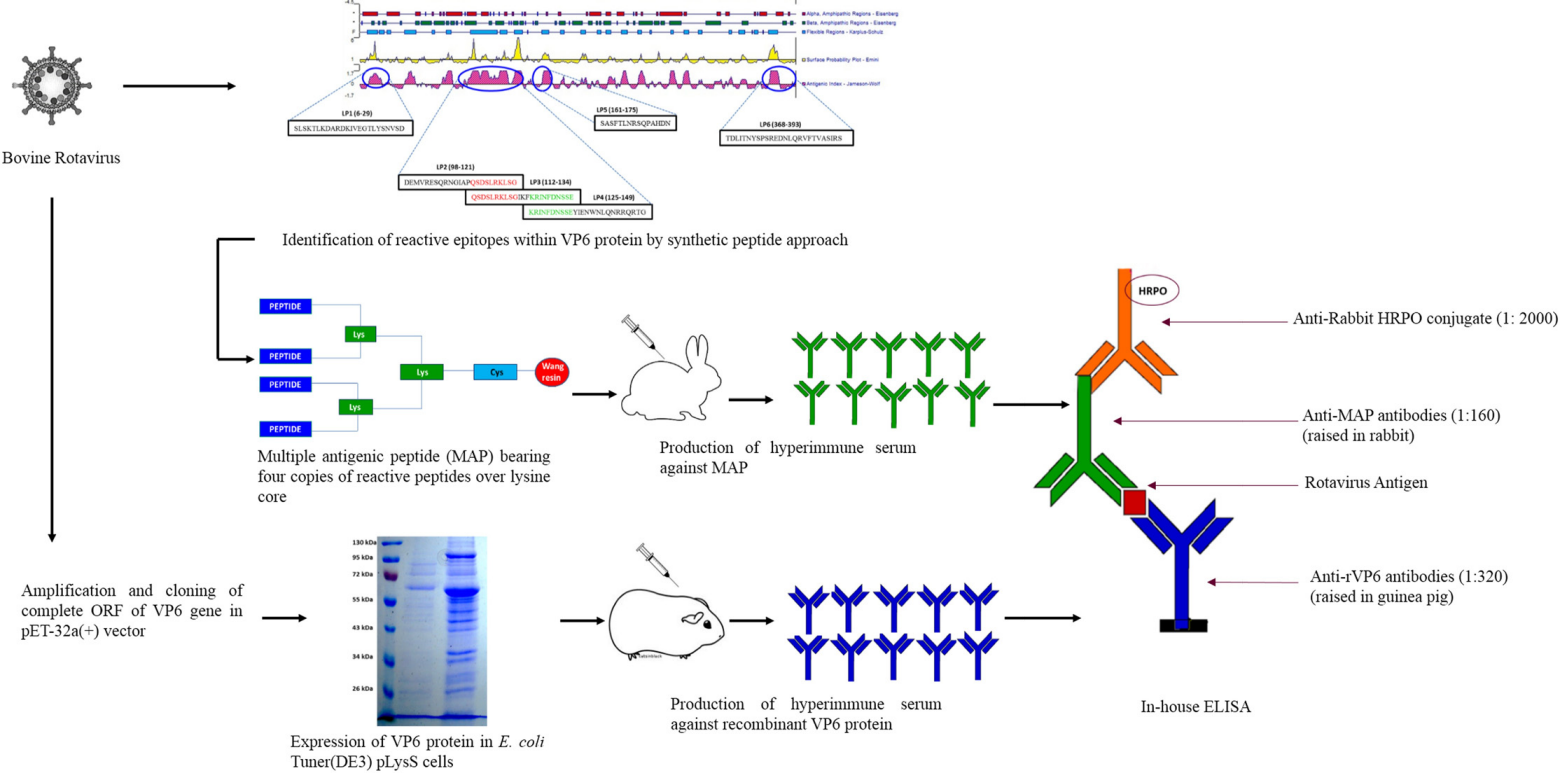
Schematic of the development process of peptide-recombinant VP6 protein based enzyme immunoassay for group A rotaviruses.

This study was undertaken to develop a novel methodology for RVAs diagnosis utilizing the potential of viral peptides. A schematic representation of the test development is provided in Fig 1. We identified the most reactive immunodominant epitopes spanning the bovine RVA VP6 protein by synthetic peptide approach. Then, multimeric peptides made up of these immunodominant epitopes in conjunction of recombinant VP6 protein were utilized for development of sensitive enzyme immunoassay capable of detecting RVA antigen in the multiple host species.

## Materials and Methods

### Ethics statement

Approval to conduct this study was obtained from the Institute Animal Ethical Committee (IAEC) [vide approval no. 1-53/2012-13/JD(R)] of Indian Council Agricultural Research-Indian Veterinary Research Institute (ICAR-IVRI), Izatnagar, Bareilly (U.P.), India. The hyperimmune sera were produced in laboratory animals in accordance with the guidelines set by the Committee for the Purpose of Control and Supervision of Experiments on Animals (CPCSEA), Ministry of Environment and Forestry, Government of India.

### Chemicals and Reagents

All reagents used for peptide synthesis were mostly dry and of HPLC grade. The Fmoc-amino acids, coupling reagent, and 2-(1H-Benzotriazole-1-yl)-oxo-1,2,3,3-tetramethyluronium hexafluorophosphate (HBTU) were obtained from GL Biochem (Shanghai, China); Wang resin was from Nova Biochem, Switzerland; and 1-Hydroxybenzotriazole (HOBt), N,N´-Diisopropylcarbodiimide (DIPC), 2,2,2-Trifluoroethanol (TFE), and N,N-Diisopropyl ethylamine (DIEA) were from Spectrochem, India.

Cloning vector pET-32a(+) and four *E*. *coli* expression strains [BL21(DE3)pLysS, Origami(DE3)pLysS, Rosetta-gami(DE3)pLysS, and Tuner(DE3)pLysS] were from Novagen (Germany). African green monkey kidney (MA-104) cell-adapted bovine RVA (positive control in assay development) and anti-RVA polyclonal serum were kindly provided by Dr. Minakshi Prasad, Department of Animal Biotechnology, Lala Lajpat Rai University of Veterinary and Animal Sciences, Hisar, Haryana, India.

### Synthesis and purification of peptides

The selection of bovine RVA (UKD-M1/09) strain for this study was based on multiple sequence alignments with public domain retrieved RVAs of different host species. The UKD-M1/09 strain is a multiple-species reassortant isolated in 2009 from a male cross-bred calf with gastroenteritis in the North-West Himalayan city of Mukteswar (Nainital), Uttarakhand, India. We identified a total of six regions within VP6 protein of this strain that showed high Jameson-Wolf antigenic index (Protean software, DNASTAR) as given in Table 1 and S1 Fig.

**Table 1:** Peptides spanning the major immunodominant epitopes within the bovine RVA VP6 protein.

Name of Peptide	Position	Length	MolecularWeight (Da)	Attribute	Amino acid sequence
P-1	6–29	24 mer	2639.92	Neutral	SLSKTLKDARDKIVEGTLYSNVSD
P-2	98–121	24 mer	2673.93	Neutral	DEMVRESQRNGIAPQSDSLRKLSG
P-3	112–134	23 mer	2669.96	Basic	QSDSLRKLSGIKFKRINFDNSSE
P-4	125–149	25 mer	3139.37	Basic	KRINFDNSSEYIENWNLQNRRQRTG
P-5	161–175	15 mer	1644.71	Basic	SASFTLNRSQPAHDN
P-6	368–393	26 mer	2983.26	Neutral	TDLITNYSPSREDNLQRVFTVASIRS

The peptides covering identified regions were manually synthesized on Wang resin (Nova Biochem, Switzerland) using Fmoc-(Nα-9-Fluorenylmethyloxycarbonyl) chemistry and solid phase peptide synthesis as per the methods described earlier [22–24]. Briefly, Fmoc-glycine was activated with N,N´-Diisopropylecarbodiimide (DIPC) and 4-Dimethylaminopyridine (DMAP) followed by its coupling with Dimethylformamide (DMF) pre-swollen Wang resin beads. The capping of free functional groups on Wang resin was done with an acetylation mixture [DMF: Acetic anhydride: N,N-Diisopropylethylamine (DIEA), (193: 6: 1, v/v)] to avoid undesired peptide synthesis. Chemical deprotection of N-terminal Fmoc group of glycine was done by 20% piperidine in DMF (v/v). Each successive Fmoc-amino acid to be used in peptides synthesis was activated by HOBt and HBTU, and coupled in the presence of DIEA. Rest steps were performed in a similar manner until the desired length of the peptide was obtained. The last deprotection (removal of side chain protecting groups) and cleavage from resin beads were done by treating the resin bound peptides with a cleavage mixture [Trifluoroacetic acid (TFA): Phenol: Thioanisol: Ether: Water (82.5: 5: 5: 5: 2.5, v/v)]. Finally, the cleaved peptide was precipitated with chilled (dry) diethyl ether, vacuum dried and stored under dry conditions until used. Purification of peptides was done by Reversed Phase- High Performance Liquid Chromatography (RP-HPLC) performed on a Shimadzu SCL-10AVP ver. 5.42 liquid chromatograph equipped with semi-preparative, RP C-18 column (Toyasoda, Japan). Linear gradients of acetonitrile in aqueous 0.1% TFA (v/v) were used to elute peptides. Peptide elution was monitored at 220nm using a SPDM10AVP photodiode array detector. Chromatograph was analyzed using inbuilt software.

### Synthesis and purification of multiple antigenic peptides (MAPs)

The MAPs were constructed on peptidyl core of three radially branched lysine residues onto which desired peptide sequences were built using step-wise Fmoc solid-phase peptide synthesis [22–23]. Cysteine was attached to Wang resin after activation in the presence of DIPC and DMAP. The capping of free functional groups on Wang resin and chemical deprotection of N-terminal Fmoc group of cysteine was done as described above. The peptide branching was achieved to form four arm structure using di-Fmoc-Lys-OH to provide lysine core over which chosen peptide sequence was synthesized (S2 Fig). Fmoc-amino acids coupling was done as described above, until the desired length of the multimeric peptide was obtained. Final chemical deprotection and cleavage were done by treating resin bound peptides with the cleavage mixture. Lastly, cleaved MAP was precipitated with chilled (dry) diethyl ether, vacuum dried and stored under dry conditions until used. The MAPs were purified by RP-HPLC.

### Circular Dichroism (CD) Spectroscopy

The CD spectra were recorded at room temperature (~20°C) in a quartz cuvette of 0.1 cm path length on a JASCO-J810 spectropolarimeter calibrated with d-camphor-10-sulfonate. The experiments were performed in polar (HPLC water) and non-polar (Trifluoroethanol) solvents to investigate the effect on conformation of both peptides and multiple antigenic peptides. The RP-HPLC purified peptides were used at a concentration of 100μg/ml. Each spectrum was recorded in a continuous scanning mode (at an average of four repeated scans with 100nm/sec scanning speed), and response time of 1 sec. The contribution of buffer/solvent was duly subtracted from each spectrum. Respective intensities were expressed in mean residue molar ellipticity [*ϑ*], calculated from the following equation:

$$[\theta ]=\frac{100\psi }{C\times L}$$

Where [*ϑ*] is molar ellipticity in deg.cm^2^.decimole^-1^, Ψ is observed ellipticity in degree, C is concentration in mole Lit^-1^, L is the path length in cm.

The line shapes of recorded spectra were analyzed using a least-square fitting routine in comparison to poly-lysine standards representing 100% α-helix, β-turn or random coil, respectively. The secondary structure estimation by Spectra Manager software provided an assessment of secondary structural elements of each peptide in solution [25].

### Peptide- ELISA (P- ELISA)

The six peptides covering the major immunodominant epitopes of bovine RVA VP6 protein were screened by P- ELISA. In brief, each peptide was coated in duplicate wells (10μg/well) of high binding 96-well polystyrene Maxisorp ELISA plate (Nunc, Denmark) in carbonate-bicarbonate buffer, pH 9.6. The plate was incubated overnight at 4°C. The unoccupied sites on the well surfaces were blocked with 200 μl of 2% bovine serum albumin (BSA) after washing three times with phosphate buffered saline (PBS, pH 7.4) and incubated at 37°C for 2 hours. The peptides bound to polystyrene surface were made to react with 50μl of rabbit anti-RVA polyclonal serum (1:100 dilutions in 2% BSA) for 1 hour at 37°C. After washing three times with PBS, the plate was incubated with 50μl of anti-rabbit HRP conjugate (1:1000 in 2% BSA) (Sigma-Aldrich, USA) for 1 hour at 37°C. The plate was washed again three times with PBS and then left for colour development at 37°C for 15 minutes after addition of 50μl of OPD (o-phenylenediamine dihydrochloride) substrate (Sigma-Aldrich, USA). The reaction was stopped using 1M sulfuric acid. Optical density was recorded at a 492nm wavelength in an ELISA reader (Biorad, Model 680).

### Multiple antigenic peptide- ELISA (MAP- ELISA)

All steps in MAP-ELISA were similar to those of P-ELISA as described above except that MAPs were used as the coating antigen instead of peptides. Each of the MAPs was coated on to duplicate wells of ELISA plate at 1μg/well concentration prepared in carbonate-bicarbonate buffer, pH 9.6. The remaining steps were followed in the same way as that of P-ELISA.

### Construction of VP6 recombinant plasmid and it’s over expression

RNA from bovine rotavirus strain (UKD-M1/09) was extracted using Quick-RNA^TM^ Mini Prep Kit (Prolab, India) followed by reverse transcription. The complete VP6 ORF was amplified using sense primer having an EcoRI site (sequence underlined); VP6_EcoRI_ F [+] (*aac*GAATTCATGGATGTCCTATACTCTTTGT) and anti-sense primer containing XhoI site (sequence underlined); VP6_XhoI_R [–] (tcagCTCGAGCTCATTTGACAAGCATGC). The PCR reaction mixture consisted of 2 μL of cDNA (2μg), 25 μL of 2X DreamTaq PCR Mater Mix (Thermo Scientific), 1 μL of each primer (10 pmol) and 21 μL of NFW. The PCR was performed at initial denaturation step of 95°C for 5 min followed by 35 cycles of 94°C for 1 min, 54°C for 1 min, 72°C for 1 min 15 sec and final extension at 72°C for 10 min. The PCR products were purified using a GeneJET^TM^ Gel Extraction kit (Thermo Scientific) and further subjected to nucleotide sequencing (SciGenom Labs Pvt. Ltd., India).

To construct VP6 recombinant plasmid, doubly (EcoRI and XhoI) digested VP6 amplicon and pET-32a(+) vector were ligated in the presence of T4 DNA ligase followed by transformation in *E*. *coli* TOP 10 F’ competent cells. The recombinant clones were confirmed by colony PCR and nucleotide sequencing. The pET-32a(+)-VP6 recombinant plasmid was transformed into four different expression hosts, e.g., BL21(DE3)pLysS, Origami(DE3)pLysS, Rosetta-gami(DE3)pLysS, and Tuner(DE3)pLysS. These transformed cells were grown in Luria Bertani (LB) broth (HiMedia, India) supplemented with 1% glucose and antibiotics [ampicillin (50μg/ml) and chloramphenicol (35μg/ml)] on shaker-cum-incubator at 37°C (180 rpm). When the optical density of the culture at 600nm reached 0.8, isopropyl beta-D-thiogalactoside (IPTG) was added at to a final concentration of 1 mM followed by incubation at different temperatures and times to optimize the induction conditions. The size and yield of 6xHis-tagged recombinant VP6 (rVP6) protein were confirmed by Sodium Dodecyl Sulphate–Polyacrylamide Gel Electrophoresis (SDS-PAGE) analysis.

### Western blot analysis and rVP6 purification under denaturing conditions

Further confirmation of rVP6 protein was done by Western blot analysis utilizing two approaches viz. polyHistidine based recombinant protein detection, and anti-RVA polyclonal serum based specific detection of recombinant proteins using a semi-dry blot system [26–27]. Briefly, rVP6 protein was subjected to SDS-PAGE and protein bands were transferred over Polyvinylidene difluoride (PVDF) membrane (MDI, India) using constant voltage of 6–8 V (0.8–1 mA/cm^2^ of the gel) for 60 min. The protein bands were allowed to react with monoclonal anti-polyHistidine peroxidase conjugate (1:1000 in 3% BSA) and rabbit anti-RVA polyclonal serum (1:500 in 3% BSA) separately, after blocking the unoccupied sites by 3% BSA at 4°C overnight. The anti-RVA polyclonal serum reacted protein bands were further incubated with anti-rabbit HRPO conjugate (1:1000 in 3% BSA) after washing with PBST (0.1% Tween-20 in PBS). The protein bands were visualized using 3,3′-Diaminobenzidine (DAB) (Sigma-Aldrich, USA).

The over-expressed 6xHis-tagged rVP6 protein formed inclusion bodies as revealed by solubility analysis. Therefore, rVP6 protein was purified by Nickel- Nitrilotriacetic acid (Ni-NTA) affinity chromatography after solubilizing the inclusion bodies in 6M guanidium hydrochloride. Briefly, an equal volume of Lysis-Equilibration (LE) buffer (50mM NaH_2_PO_4_, 300mM NaCl, 8M Urea, pH-8.0) with guanidium hydrochloride treated cell lysate was allowed to pass through Ni-NTA agarose column after equilibration. The column was washed with five bed volumes of eight wash buffers (50mM NaH_2_PO_4_, 300mM NaCl, 30mM Imidazole, 7 to 0 M Urea, pH-8.0) using decreasing urea concentration from 7M to 0M in each of the subsequent wash buffers. The rVP6 protein was finally eluted with two bed volumes of elution buffer (50mM NaH_2_PO_4_, 300mM NaCl, 250mM Imidazole, pH-8.0). The flow through collected at each step were analyzed by SDS-PAGE. After purification, rVP6 protein was dialyzed overnight against PBS (pH, 7.4) at 4°C by using cellulose membrane (12 kDa) (Sigma-Aldrich, USA) and quantified spectrophotometrically at 280 nm using Nanodrop (ND-1000, Thermo-Scientific, USA).

### Generation of hyperimmune serum in rabbits and guinea pigs

Four New Zealand white rabbits (females aged 4 months and weighing 2.5–3.0 kg) and six guinea pigs (females aged 6 months and weighing 600–700 gm) were procured from Laboratory Animal Resources (LAR), ICAR-IVRI, Bareilly, Uttar Pradesh, India. All the guidelines of CPCSEA were strictly followed to minimize stress and discomfort and the animals were monitored daily for any disease condition. Pre-immunization serum was collected aseptically from the marginal ear vein of rabbits and saphenous vein of guinea pigs. Emulsion preparations consisting of an equal volume of antigens (250 μg of antigen/rabbit, and 100 μg of antigen/guinea pig) and Freund’s complete adjuvant (FCA) were formulated. Two rabbits each were immunized with an emulsion prepared from MAP and rVP6 protein by subcutaneous route at 4–6 sites (0.2 ml/site). Three guinea pigs each were inoculated with an emulsion prepared from MAP and rVP6 protein by subcutaneous route at 3–5 sites (0.2 ml/site). Subsequent boosters at 21^th^, 28^th^ and 35^th^ days post-first immunization were administered using an emulsion made of an equal volume of antigens and Freund’s incomplete adjuvant (FIA). Finally, exsanguination was performed on 42^nd^ day post-first immunization through cardiac puncture after administration of general anesthesia in rabbit [Xylazine (5 mg/kg) + ketamine (30 mg/kg)] and guinea pig [Xylazine (2 mg/kg) + ketamine (80 mg/kg)] by the intramuscular route followed by pentobarbital overdose in accordance with CPCSEA. The sera after extraction from blood were stored at -20°C till further use.

### Development and validation of sandwich ELISA for RVA antigen detection

Hyperimmune sera generated against rVP6 and MAP were used as capture and detector antibodies, respectively and also *vice-versa*. Optimal dilutions of the detector and capture antibodies from both animal species were determined by standard checkerboard titrations. Sandwich ELISA was optimized as follows: 50 μl of guinea pig anti-rVP6 antibodies (1:320 in carbonate-bicarbonate buffer, pH 9.6) were coated onto high binding 96 well polystyrene Maxisorp ELISA plates (Nunc, Denmark) and kept at 4°C overnight. Next day, the wells were incubated with 200 μl blocking buffer (5% Bovine serum albumin and 5% Skimmed milk powder in the ratio of 1:1) at 37°C for 2 hrs, after three washes with 0.1% Tween-20 in PBS (PBST). The wells were incubated with 50 μl of processed fecal samples (10% suspensions made in PBS) in duplicate at 37°C for 1 hr after one time wash with PBST. Then, the wells were incubated with rabbit anti-MAP antibodies (1:160 in blocking buffer) at 37°C for 1 hr, after three washes with PBST. Finally, 50 μl of anti-rabbit HRP conjugate (Sigma-Aldrich) diluted to 1:2000 in blocking buffer was transferred in each well and incubated at 37°C for 45 min. The optical density (OD_492_) values were recorded in an ELISA reader after the addition of OPD (o-phenylenediamine dihydrochloride) (50μl/well) for 10 min at 37°C followed by stopping the reaction with 1M H_2_SO_4_. The OD_492_ was recorded for each of the samples in duplicate.

To reduce variations due to differences in absolute absorbance values obtained at each run, the OD for each test sample was expressed as a percent of the positive control (OD_P_) using the formula:

$$\text{Percent positivity}\;\left(\mathrm{PP}\right)\;\text{value}=(\text{O}{\text{D}}_{\text{S}}-\text{O}{\text{D}}_{\text{CC}})/(\text{O}{\text{D}}_{\text{P}}-\text{O}{\text{D}}_{\text{CC}})\;\text{x}\;100$$

Where OD_S_ is the OD of test samples, OD_CC_ is the OD of conjugate control, OD_P_ is the OD of positive control.

For determination of cut-off values, 40 each of RVA positive and negative samples from each of the species were tested in triplicate in two separate experiments. The end-point cut off (in terms of PP value) was estimated by Receiver Operating Characteristic (ROC) curve analysis so as to achieve maximum diagnostic sensitivity (DSn) and diagnostic specificity (DSp). In addition, the specificity of the optimized in-house ELISA was checked with other enteric (rotavirus group B, rotavirus group C, picobirnavirus) and non-enteric (bluetongue virus) viruses.

For estimating the repeatability of the assay, we tested eight each of RVA positive and negative fecal samples having different PP values. For intra-assay (within-plate) repeatability, three replicates of the same fecal sample were tested in the same ELISA plate. The inter-assay (between-run) repeatability was performed by two technicians in two different labs by testing each sample in triplicate.

### Comparison of three diagnostic assays (RNA-PAGE, diagnostic RT-PCR and in-house ELISA) and commercially available ELISA diagnostic kits

A total of 914 diarrhoeic fecal samples collected from different species [bovine (n = 368); porcine (n = 317); human (n = 111) and poultry (n = 118)] from different geographical regions of India were screened for the presence of RVA antigen by three diagnostic assays (i.e. RNA-PAGE, diagnostic RT-PCR and in-house ELISA) (S1 Table). Briefly, RNA-PAGE was performed in 10% polyacrylamide gel using the discontinuous buffer system [28] and genome segments were visualized by silver staining [29–30]. The diagnostic RT-PCR was performed using self-designed primers targeting the conserved region of VP6 gene; VP6_F (885–902) [+] (5´-ACGWCCACCRAATATGAC-3´) and VP6-R (979–1000) [–] (5´-GATTCACAAACTGCAGATTCAA-3´). The PCR reaction mixture contained 2 μL of cDNA (2μg), 25 μL of 2X DreamTaq PCR Mater Mix (Thermo Scientific), 1 μL of each primer (10 pmol) and 21 μL of NFW. Reaction conditions were: 35 cycles of 5 min at 95°C, 30 sec at 94°C, 10 sec at 46°C, 10 sec at 72°C and final extension at 72°C for 5 min. The in-house sandwich ELISA was performed as described elsewhere in the manuscript.

Further, we compared in-house ELISA with commercially available diagnostic kits (IDEXX Rotavirus ELISA kit and Bio-X Diagnostics ELISA kit). Randomly chosen bovine (n = 39), and small ruminants (sheep and goat) (n = 25) fecal samples were screened for the detection of RVA antigen and compared with the IDEXX Rotavirus ELISA kit and Bio-X Diagnostics ELISA kit, respectively.

### Data analysis

ROC curve analysis was performed by MedCalc® statistical software 13.1.1.0 [31] where true RVA positive and negative samples were assigned the values of 1 and 0, respectively. The area under the ROC curve (AUC) was then calculated for samples from each of the species. The AUC values close to 1 indicated a highly precise test [31]. The cut-off points were also determined using ROC curve analysis for each species.

The Chi-square test was used to analyze the significance of differences among three diagnostic assays (RNA-PAGE, in-house ELISA and RT-PCR) in their ability to differentiate RVA positive and negative samples, and was performed by XLSTAT Version 2015 and GraphPad Prism 6 (GraphPad Software, San Diego, California, USA). Further, the concordance of the three assays was estimated by inter-rater reliability analysis using Kappa (κ) statistic [32]. Values of κ< 0 indicate poor agreement; 0.00 to 0.20, slight agreement; 0.21 to 0.40, fair agreement; 0.41 to 0.60, moderate agreement; 0.61 to 0.80, substantial agreement and 0.81 to 1.00, perfect agreement [32].

## Results

We identified six regions of high antigenic index within bovine RVA VP6 protein and correspondingly synthesized peptides by solid phase peptide synthesis over Wang resin. Purity of all peptides was greater than 85% as determined in semi-preparative RP-HPLC.

### Characterization of peptides by CD spectroscopy

**Fig 2:**
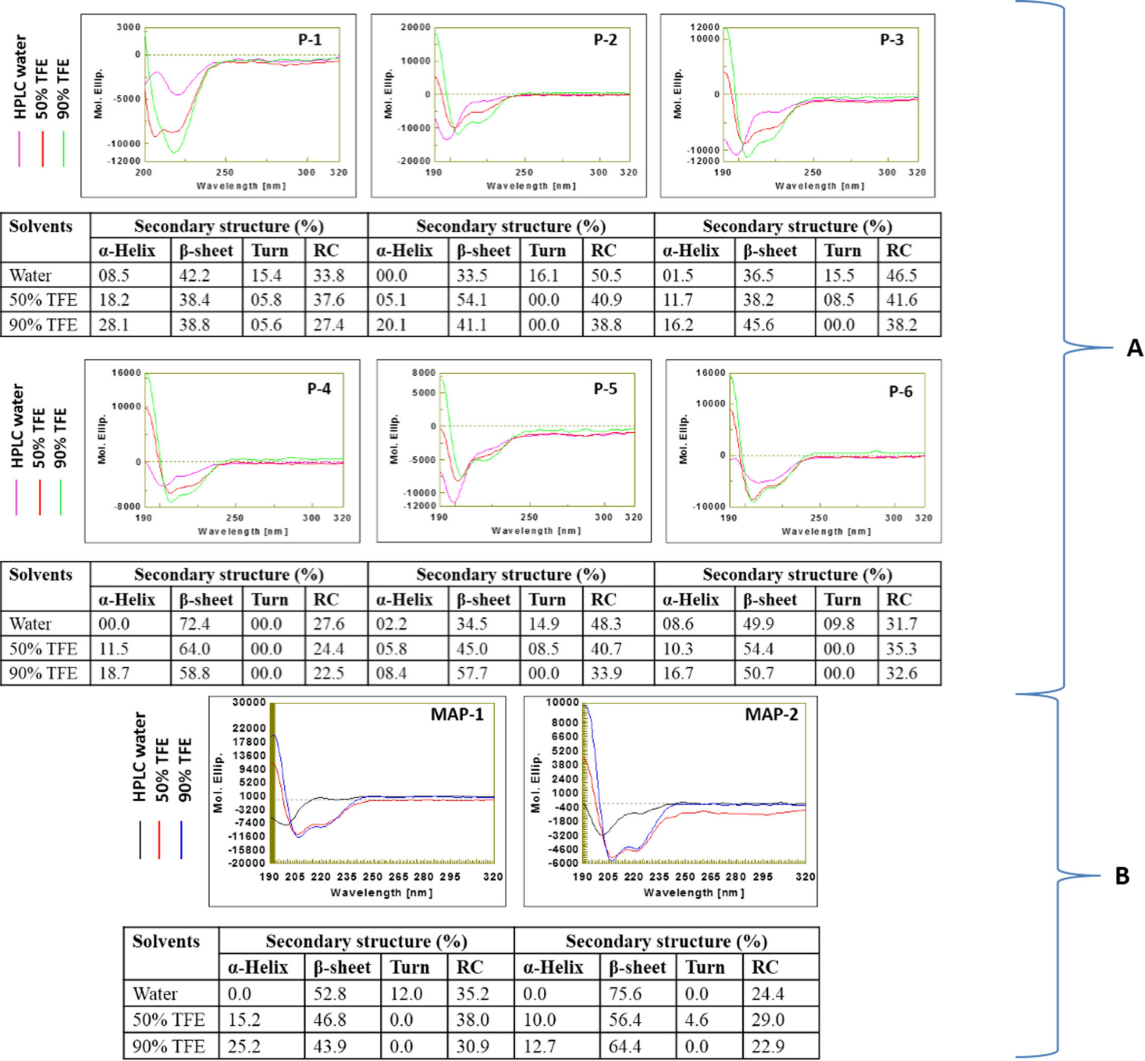
The CD spectra of (A) peptides (P1 –P6) and (B) multiple antigenic peptides (MAP-1, and MAP-2) in different solvents (HPLC water, 50% TFE and 90% TFE). (Secondary structure compositions of peptides in different solvents are also provided in the respective tabular form, RC- random coil).

The behavior of peptides and MAPs were examined in both polar and non-polar solvents. The CD spectra analysis revealed that all six peptides in polar solvent had a negative minimum below 200 nm in a far UV range. Changing the environment from polar to non-polar [HPLC water to Trifluoroethanol (TFE)] resulted in a decline in ellipticity at 197nm followed by increase in negative ellipticity around 220nm for all the peptides, which is generally credited to the formation of more ordered conformation [33]. We further analyzed secondary structure compositions of all peptides in different environments. In polar solvent, the disordered conformation predominated in all six peptides (Fig 2A). Addition of non-polar solvent (50% TFE) led to induction of ordered structures. Further increase in non-polar solvent concentration to 90% TFE led to additional improvement in the ordered structures of all six peptides. We observed a peculiar secondary structure composition of P4 peptide i.e. total ordered conformations of P4 in polar and non-polar solvents were 72.4% and 77.5%, respectively (Table 2).

**Table 2:** Behavior of peptides in polar and non-polar solvents as determined by CD spectroscopy.

Peptides	Polar solvent (HPLC water)		Non-polar solvent (90% TFE)	
	*Ordered (%)*	*Disordered (%)*	*Ordered (%)*	*Disordered (%)*
P1	50.7	49.2	66.9	33.0
P2	33.5	66.6	61.2	38.8
P3	38.0	62.0	61.8	38.2
P4	72.4	27.6	77.5	22.5
P5	36.7	63.2	66.1	33.9
P6	58.5	41.5	67.4	32.6
MAP1	52.8	47.2	69.1	30.9
MAP2	75.6	24.4	77.1	22.9

On analyzing the CD spectra of MAPs, interestingly, β-sheet composition dominated in both MAPs in the polar solvent i.e. 52.8% β-sheet in MAP-1 (four arm copy of P-3) and 75.6% β-sheet in MAP-2 (four arm copy of P-4) (Fig 2B). Similar to peptides, both MAPs displayed ordered structures in non-polar environment. Total ordered conformations of MAP-2 in both polar (75.6%) and non-polar (77.1%) solvents were higher than those of MAP-1 (Table 2).

### Reactivity of peptides with rabbit anti- RVA polyclonal serum

**Fig 3:**
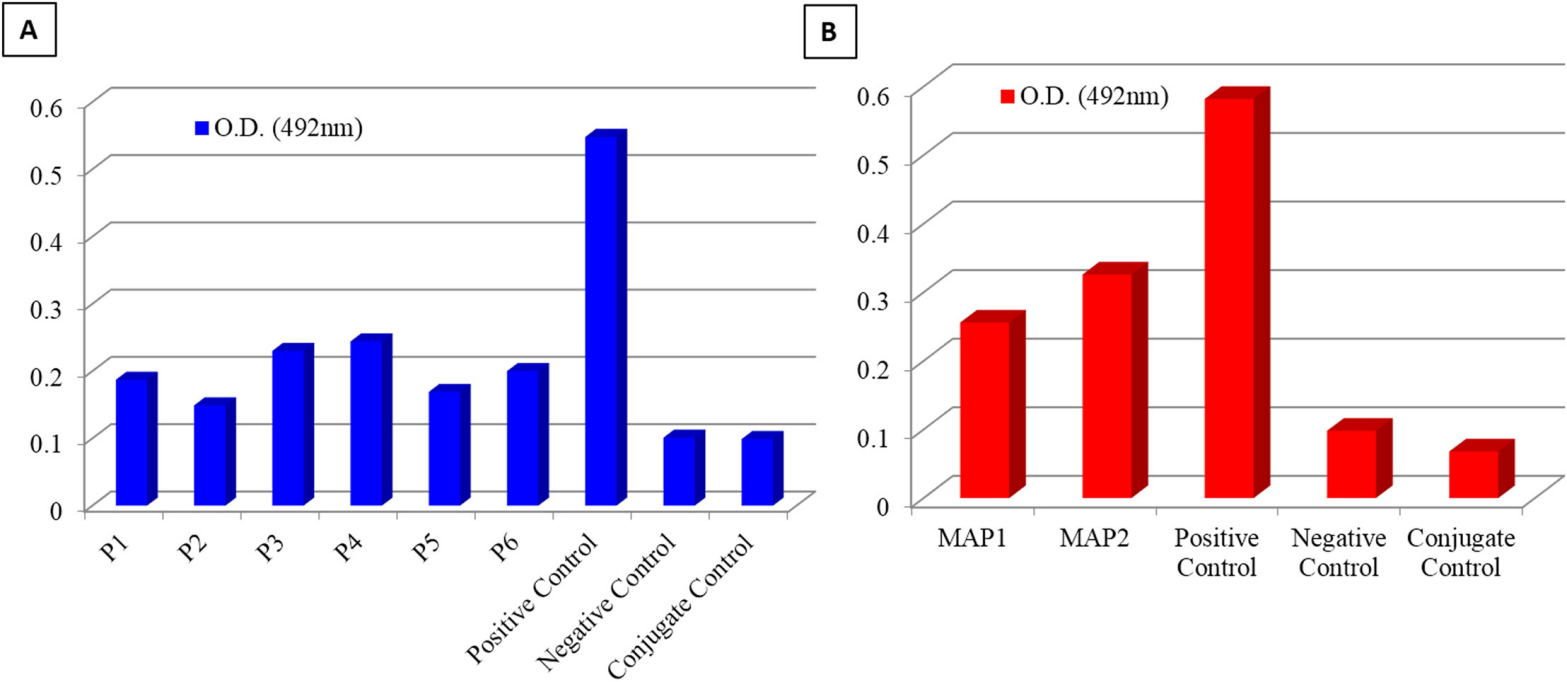
Reactivity of peptides (A) and multiple antigenic peptides (B) with rabbit anti- RVA polyclonal serum in indirect ELISA format.

Reactivity of all peptides was evaluated in an indirect ELISA. Two peptides synthesized from an overlapping region, P-3 (QSDSLRKLSGIKFKRINFDNSSE-Gly; 112-134aa) and P-4 (KRINFDNSSEYIENWNLQNRRQRTG-Gly; 125-149aa) were reactive with anti- RVA polyclonal serum (Fig 3A). Since peptides are less immunogenic because of low molecular weight, four arms copy of these peptide sequences (P-3 and P-4) were synthesized separately on lysine mosaic prepared on Wang resin. Reactivity levels of MAP-2 [(KRINFDNSSEYIENWNLQNRRQRTG)_4_-Lys_2_-Lys-Cys] was higher in comparison to that of MAP-1 [(QSDSLRKLSGIKFKRINFDNSSE)_4_-Lys_2_-Lys-Cys] in an indirect ELISA (Fig 3B), indicating that the region spanning 125-149aa within VP6 protein of bovine RVA was highly reactive with rabbit anti- RVA polyclonal serum. Hence, MAP-2 was used for generation of hyperimmune serum in laboratory animals.

### VP6 protein: Over-expression, purification and characterization

**Fig 4:**
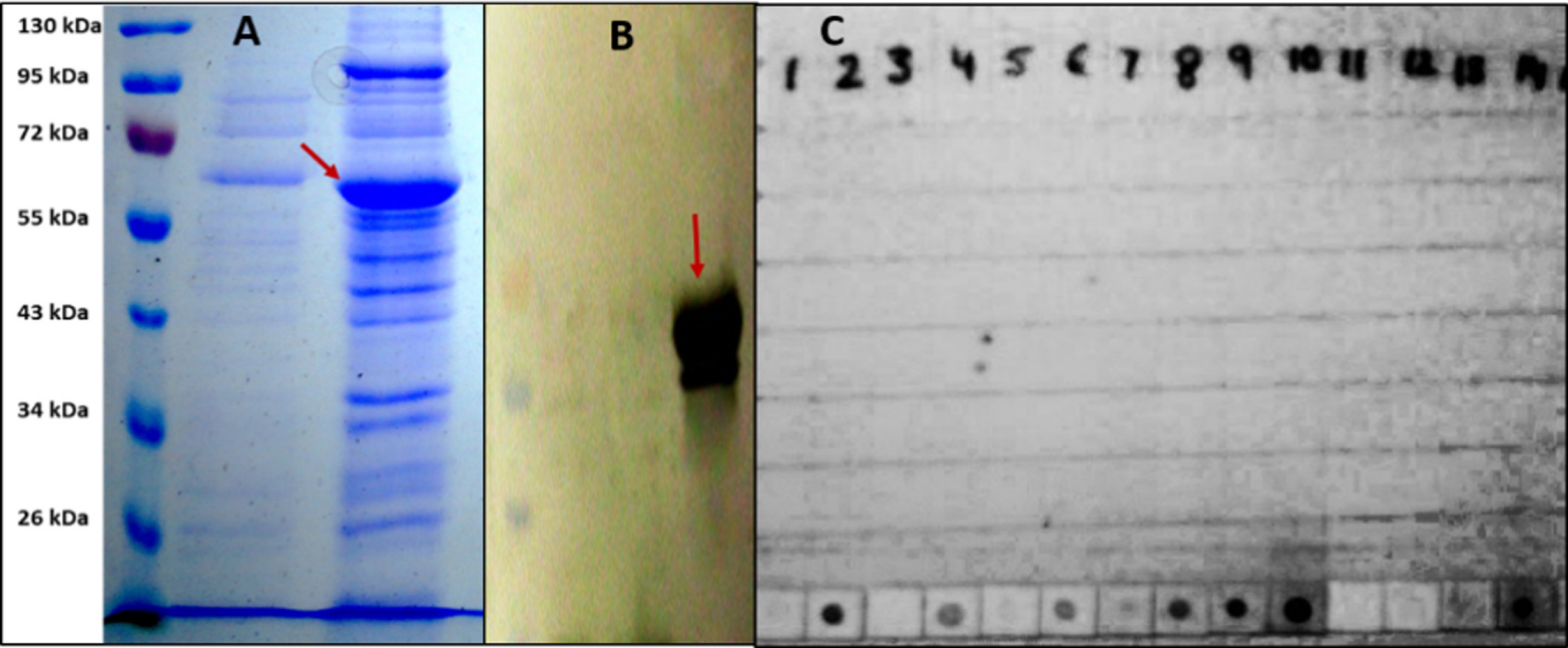
(A) Coomassie-blue stained SDS–PAGE of cell lysates with (indicated by red arrow) and without IPTG-induction in expression host [*E*. *coli* Tuner(DE3)pLysS cells], (B) Western blot analysis of recombinant hexahistidine-tagged VP6 fusion protein of bovine RVA on polyvinyl difluoride (PVDF) membrane (indicated by red arrow), (C) Dot blot assay for detection of RVA antigen in bovine fecal samples using anti-rVP6 antibodies.

Of the four expression hosts evaluated, Tuner(DE3)pLysS cells displayed the highest level of expression of recombinant VP6 protein (rVP6) (Fig 4A). This expression level was approximately 1.5 fold higher compared to Rosetta-gami(DE3)pLysS cells as measured in SDS-PAGE analysis. Expression levels in other two hosts were very low. Solubility analysis revealed the formation of inclusion bodies by over-expressed rVP6 protein. The inclusion bodies were first solubilized in 6M guanidium hydrochloride followed by allowing the folding of denatured rVP6 protein at a constant rate by decreasing the concentration of urea (8M to 0M) from the washing buffer step-by-step by Immobilized-Metal Affinity Chromatography (IMAC). Thus, we allowed two processes (washing to get rid of undesired proteins and refolding of denatured protein) to occur at the same time in Ni-NTA resin column. A total yield of ~16 mg/liter was obtained after purification under denaturing conditions.

The presence of purified rVP6 protein resolved in SDS-PAGE was confirmed by both rabbit anti- RVA polyclonal serum as well as monoclonal anti-polyHistidine peroxidase conjugate, both of which produced an intense brown color reaction with the protein size corresponding to ~62 kDa for VP6 protein (Fig 4B). Further, competence of anti-rVP6 antibodies in detecting RVA antigen in different bovine fecal samples was evaluated in dot-blot assay (Fig 4C).

### Development and validation of sandwich ELISA

**Fig 5:**
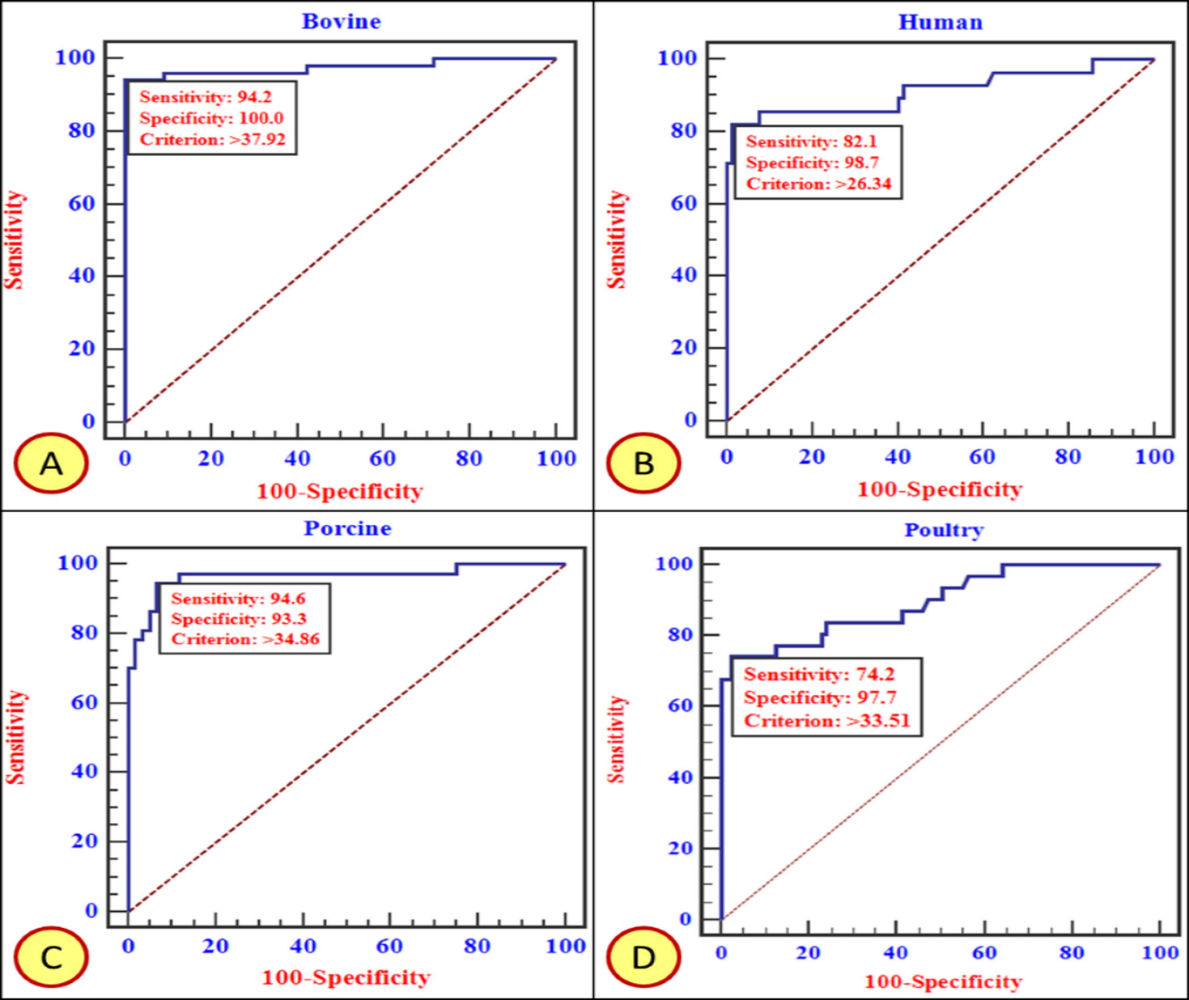
ROC curve analysis based on in-house ELISA test results obtained on screening of fecal samples from Bovine (A), Human (B), Porcine (C), and Poultry (D). Every species specific curve is provided with DSn, DSp and cut-off threshold. The area enclosed under the curve with diagonal base represents Area Under Curve (AUC).

An initial set of experiments were performed to determine the optimal concentration of the coating and detector sera. The DSn and DSp of in-house ELISA at a particular cut off values (PP values) were estimated using ROC curve analysis (Fig 5 and Table 3).

**Table 3:** Diagnostic specificity (DSp) and sensitivity (DSn) of in-house ELISA for RVA antigen detection in multiple host species.

S.No.	Species	PP cut off value (%)	DSn (%)	DSp (%)
1.	Bovine	37.92	94.2	100
2.	Porcine	34.86	94.6	93.3
3.	Poultry	33.51	74.2	97.7
4.	Human	26.34	82.1	98.7

We observed no cross-reactivity with other enteric and non-enteric viruses, ascertaining its specificity for RVA only. The intra-assay coefficient of variation (CV) of eight well-defined RVA positive samples ranged from 1.02% to 3.33%, while those of eight RVA negative samples ranged from 0.02% to 4.91%. The inter-assay CV for RVA positive samples (n = 8) was between 0.36% and 41.79%, whereas the CV for RVA negative samples (n = 8) was between 0.49% and 36.01%. It is evident that this assay is repeatable and yielded a low and acceptable variation.

Further, we evaluated the performance characteristics of three diagnostic tests (RNA-PAGE, in-house ELISA, and RT-PCR) for detection of RVA antigen in the fecal samples of four species, bovine (n = 368), porcine (n = 317), poultry (n = 118) and human (n = 111) (Table 4).

**Table 4:** Comparison of RNA-PAGE, in-house ELISA and RT-PCR for RVA antigen detection in multiple host species.

Species tested	RNA-PAGE	In-house ELISA	RT-PCR
Bovine (n = 368)	115/386 (31.25%)	143/386 (38.85%)	154/368 (41.84%)
Porcine (n = 317)	25/317 (7.88%)	120/317 (37.85%)	125/317 (39.43%)
Poultry (n = 118)	8/118 (6.80%)	23/118 (19.50%)	30/118 (25.42%)
Human (n = 111)	29/111 (26.12%)	30/111 (27.02%)	34/111 (30.63%)

Results of Chi-square test suggested that the frequency of RVA positive samples by either of the diagnostic assay combinations were different at a highly significant level (p< 0.001). We observed severe deviation in terms of RVA positivity shown by RNA-PAGE in comparison to in-house ELISA and RT-PCR, especially in porcine and poultry samples. The agreement among these three diagnostic tests was estimated by inter-rater reliability analysis based on Kappa values (κ) and results are presented in Table 5. The performance of in-house ELISA with respect to RT-PCR disclosed a perfect agreement (κ values > 0.81) for all the species tested. However, other combinations of tests showed fair to perfect agreements in different host species.

**Table 5:** Inter-rater reliability analysis among three diagnostic tests for RVA detection in multiple host species.

Measurement of Agreement	Bovine	Human	Porcine	Poultry
**RNA-PAGE vs In-house ELISA**				
Weighted Kappa (κ)	0.770	0.794	0.233	0.462
Standard error	0.0481	0.0455	0.0765	0.109
95% CI	0.675–0.864	0.705–0.883	0.0829–0.383	0.248–0.676
**In-house ELISA vs RT-PCR**				
**Weighted Kappa (κ)**	**0.955**	**0.912**	**0.956**	**0.831**
Standard error	0.0223	0.0429	0.0305	0.0612
95% CI	0.911–0.999	0.828–0.996	0.896–1	0.711–0.951
**RT-PCR vs RNA-PAGE**				
Weighted Kappa (κ)	0.794	0.845	0.215	0.352
Standard error	0.0455	0.0562	0.0721	0.0949
95% CI	0.705–0.883	0.735–0.955	0.0742–0.357	0.166–0.538

Furthermore, comparative performance of in-house ELISA revealed three and one RVA positive samples in bovine, and sheep and goat species, respectively, which were in complete concordance with the two commercial kits evaluated (Table 6).

**Table 6:** Comparative performance of in-house ELISA with commercially available diagnostic kits for RVA antigen detection.

Species	No. of samples	In-house ELISA	IDEXX Rotavirus ELISA	Bio-X Diagnostic ELISA
Bovines	39	3	3	-
Sheep & Goat	25	1	-	1

## Discussion

The development of a high sensitive and specific sandwich ELISA is described for the detection of RVA antigen in multiple host species using a novel peptide-recombinant protein approach. The commonly used commercially available diagnostic kits for RVA detection use either anti-RVA polyclonal serum or a combination of anti- VP6 monoclonal antibodies and anti- RVA polyclonal serum. The unique combination of anti-rVP6 antibodies as coating and anti-MAP antibodies as detector antibodies is novel; diagnostic use of peptides and their multimeric form has also not been documented so far in RVAs.

Previously, rVP6 protein was used either for sero-diagnosis of RVA infection in humans [34–35] or for detection of human RVA antigen using anti-rVP6 antibodies in latex agglutination test format [36]. In latex-agglutination test, antibodies against the conserved N-terminal portion of the VP6 (1–245aa) displayed high sensitivity (98.5%) and specificity (100%), which were calculated by comparing with less sensitive RNA-PAGE [36]. Recently, another study utilized anti-rVP6 antibodies in differentiation of porcine rotaviruses from other porcine viruses, but lacked validation part of the assay [37]. The major limitation of all these assays was specific-specific usage only.

To overcome these limitations, we exploited the peptide-recombinant protein approach. We identified a region spanning 125-149aa within the conserved VP6 protein of bovine RVA as highly reactive with rabbit anti- RVA polyclonal serum. Previous studies on protective epitopes mapping of RVA VP6 protein were limited to c-terminal. For examples, the region of the VP6 protein spanning from amino acids 197 to 397 has been identified to contain multiple protective epitopes (227–244aa, 232–261aa, 249–277aa, 283–307aa, 368–397aa) using a library of 11 overlapping peptides in H-2d BALB/c mice [38–39]. However, a 14 mer peptide covering 289-302aa region provided almost complete protection against EDIM challenge in BALB/c mice [39]. In another study, a VP6 epitope (amino acids, 242–259) was identified as immunodominant CD4^+^T cell epitope following intranasal immunization of BALB/c mice [40]. Though, 368–397aa region provided protection against challenge in a mouse model in the earlier study, this region did not react well with rabbit anti- RVA polyclonal serum in our study.

Further, we tried to explain as why a region spanning 125-149aa of VP6 protein was highly immune-reactive by studying the behavior of peptides of this region (P-4 and MAP-2) in polar and non-polar solvents. It has been suggested that antibodies prefer binding to ordered region of antigen and that more avidity is seen with an increase in ordered conformation [41]. Hence, high immune-reactivity of P-4 peptide with anti-RVA polyclonal serum might be triggered by the following possible reasons: (i) more uniform change in the ordered structures with an increase in non-polar solvent concentration; (ii) high ordered conformation in both polar (72.4%) and non-polar (77.5%) solvents; (iii) corresponding decrease in % β-sheet structure on increasing the non-polar solvent concentration; or (iv) lower negative molar ellipticity value (< –8000 deg.cm^2^.decimole^-1^) compared to other peptides. Comparatively high immune-reactivity of MAP-2 with anti-RVA polyclonal serum may be explained by high ordered secondary structure in both polar (75.6%) and non-polar (77.1%) solvents or by, lower negative molar ellipticity value for MAP-2 (< –8000 deg.cm^2^.decimole^-1^) compared to MAP-1.

We expressed full length VP6 recombinant protein in prokaryotic expression system. Our goal was to produce mono-specific antibodies that react with diverse RVA strains. The eukaryotic expression system is usually preferred for full expression of VP6 mainly because of the inhibitory effect of recombinant VP6 on *E*. *coli* growth leading to low yield [42–44]. Even, some researchers have used codon optimized VP6 protein to achieve high yield [45]. However, we were successful in achieving a significantly high yield of VP6 protein in *E*. *coli* Tuner(DE3) pLysS cells under optimized induction conditions without codon optimization.

Peptide-recombinant protein approach exploited capture antibodies against rVP6 protein (thus avoiding the use of whole virus antigen which is more prone to yield false reactions) and detector antibodies against MAP-2 (more or less equivalent to monoclonal antibodies) to achieve high DSn and DSp. However, comparatively lower DSn especially in poultry samples might be due to low RVA excreted from their faeces. It is notable that concordance between in-house ELISA and RT-PCR tests performance (κ values close to 1) was almost in perfect agreement in all the species evaluated in comparison to other combinations of tests. The RT-PCR, no doubt is a highly sensitive test, still ELISA is preferred for RVA antigen detection because results of ELISA correlate well with the clinical disease [46–47]. Also, use of enzyme immunoassay has been suggested for vaccine efficacy evaluations in patients with acute gastroenteritis [46].

Recently, quantitative real time RT-PCR has been developed to distinguish symptomatic infections from asymptomatic based on the threshold cycle cut-off values [47], but again the cost involved might restrict its usage on a large scale especially in the low income settings. The performance of most of the RVA diagnostic tests is concentrated from well-resourced settings [48–49]. However, the pattern of RVA-associated gastroenteritis in developing countries differs from that in developed countries in terms of high levels of infection, early disease onset, and delayed acquisition of immunity, which may impact accurate and prompt diagnosis [50–51]. Based on the comparable performance of our test with the two commercial ELISA kits together with the results of inter-rater reliability assay, our test might be more suitable as a preliminary assay in RVA surveillance studies, especially in resource poor countries.

In conclusion, peptide-recombinant protein approach using anti-rVP6 antibodies as coating and anti-MAP2 antibodies as detector antibodies in sandwich ELISA format provides a potential diagnostic assay for RVA antigen detection in multiple host species. This assay has a potential use in surveillance studies and in monitoring vaccine effectiveness in low and middle income settings. Adaptation of the newly developed reagents to a rapid-antigen test format would be ideal since no additional equipment would be needed.

## Supporting Information

S1 Fig Identification of regions within bovine RVA VP6 protein showing high antigenic index by bioinformatics tool. (TIF)

S2 Fig Schematic representation of Multiple Antigenic Peptide (MAP) design. (TIF)

S1 Table Details of fecal samples collected from different species in diverse geographical locations of India. (DOCX)
