# Supplementary figures and images for: Peptide-Recombinant VP6 Protein Based Enzyme Immunoassay for the Detection of Group A Rotaviruses in Multiple Host Species
Source: PLoS One. 2016 Jul 8;11(7):e0159027. doi: 10.1371/journal.pone.0159027 (PMC4938596; doi:10.1371/journal.pone.0159027)

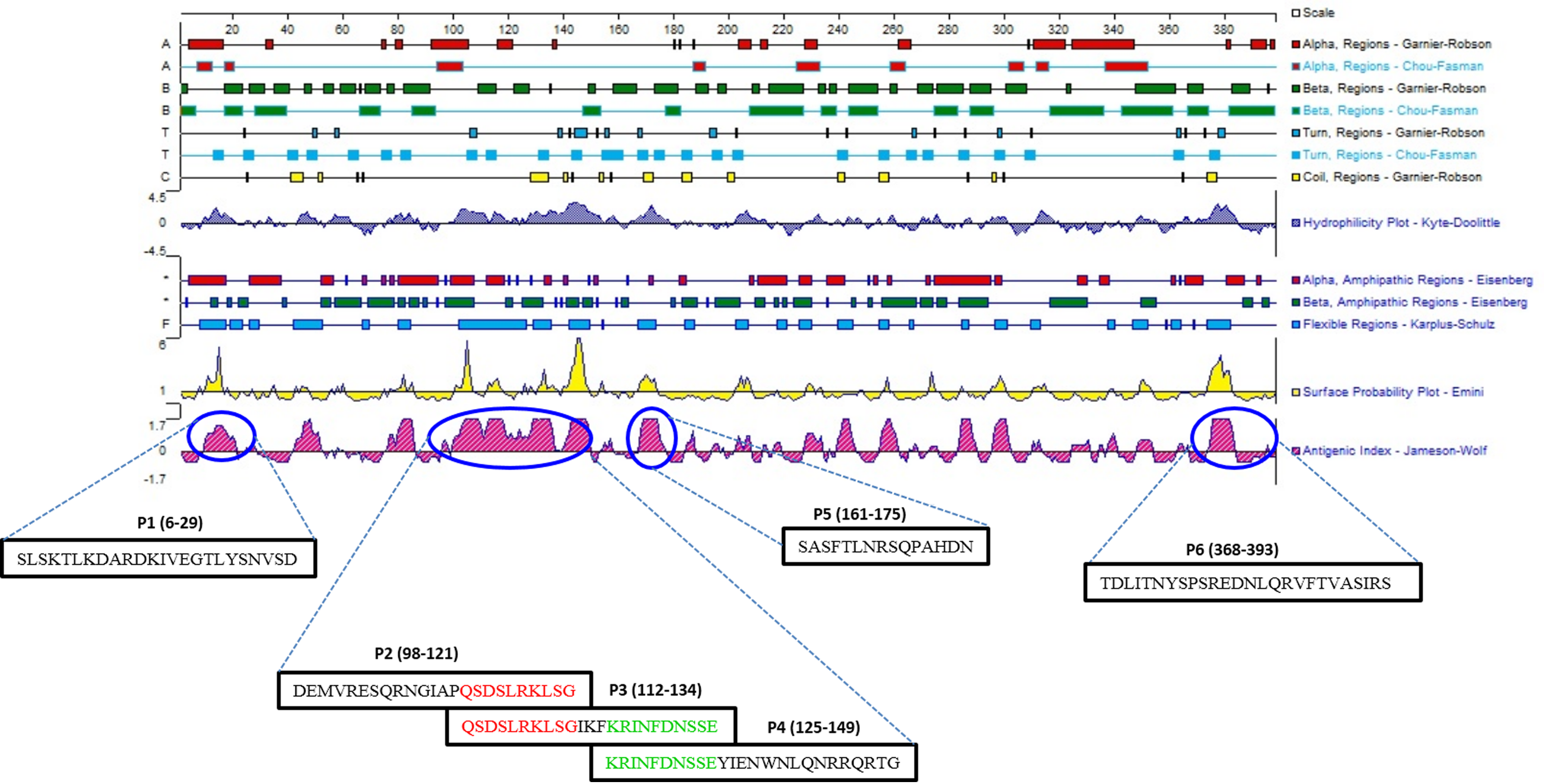

Supplement: S1 Fig — (TIF) [file pone.0159027.s001.tif]

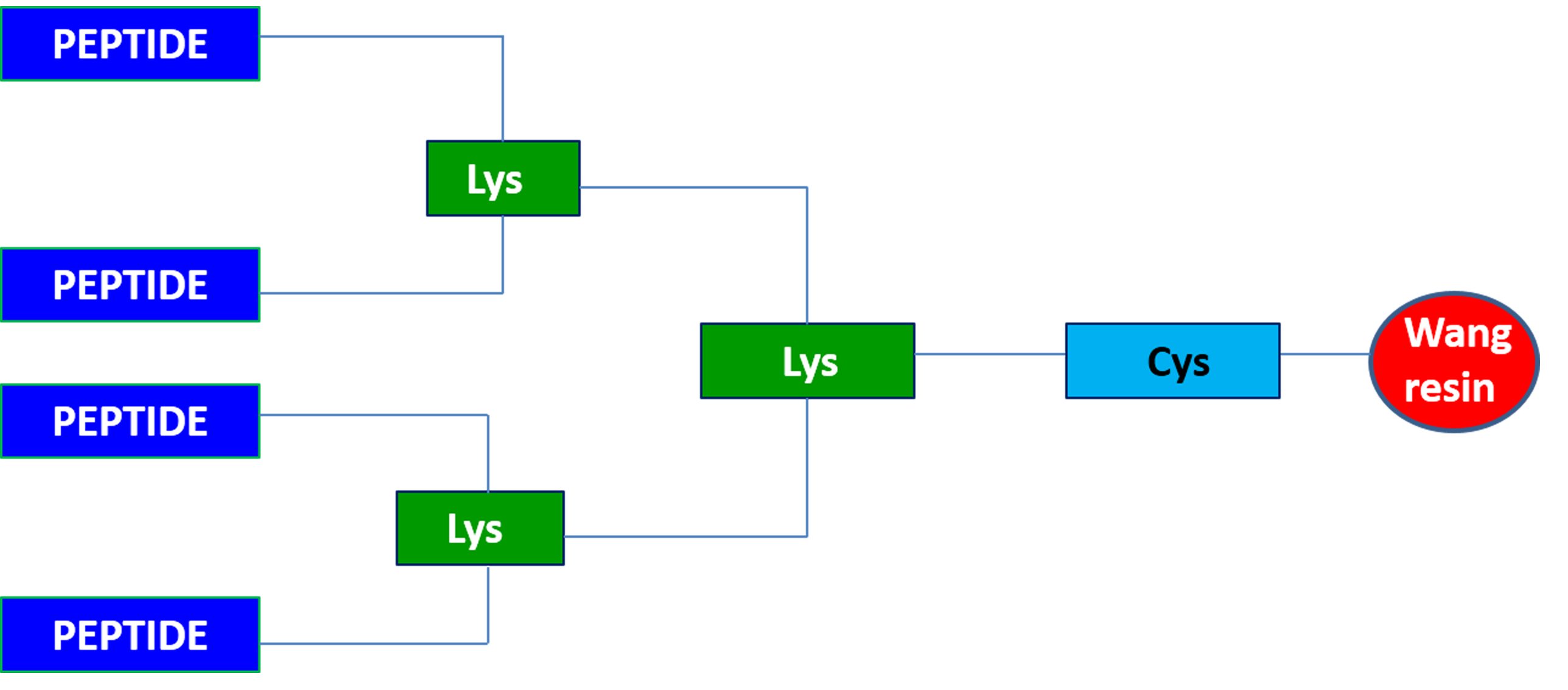

Supplement: S2 Fig — (TIF) [file pone.0159027.s002.tif]
