# Supplementary material for: Peptide-Recombinant VP6 Protein Based Enzyme Immunoassay for the Detection of Group A Rotaviruses in Multiple Host Species
Source: PLoS One. 2016 Jul 8;11(7):e0159027. doi: 10.1371/journal.pone.0159027 (PMC4938596; doi:10.1371/journal.pone.0159027)
Supplement: S1 Table — (DOCX) [file pone.0159027.s003.docx]

**S1 Table. Details of fecal samples collected from different species in diverse geographical regions of India**

| **S. No.** | **Species** | **Place of sample collection** | **No. of faecal samples** |
| --- | --- | --- | --- |
|  | Bovine | Haryana | 229 |
|  |  | Uttarakhand | 128 |
|  |  | Uttar Pradesh | 11 |
|  | Human | Uttarakhand | 111 |
|  | Porcine | North-Eastern Region | 289 |
|  |  | Uttar Pradesh | 28 |
|  | Poultry | Uttarakhand | 71 |
|  |  | Haryana | 47 |
| **Total samples** | | | **914** |
